# Increased Concentrations of Insulin-Like Growth Factor Binding Protein (IGFBP)-2, IGFBP-3, and IGFBP-4 Are Associated With Fetal Mortality in Pregnant Cows

**DOI:** 10.3389/fendo.2018.00310

**Published:** 2018-06-12

**Authors:** Kirsten Mense, Julia Heidekorn-Dettmer, Elisa Wirthgen, Yette Brockelmann, Ralf Bortfeldt, Sarah Peter, Markus Jung, Christine Höflich, Andreas Hoeflich, Marion Schmicke

**Affiliations:** ^1^Institute for the Reproduction of Farm Animals Schoenow, Bernau, Germany; ^2^Clinic for Cattle, University of Veterinary Medicine Hannover, Foundation, Hannover, Germany; ^3^Institute of Genome Biology, Leibniz Institute for Farm Animal Biology, Dummerstorf, Germany; ^4^Ligandis UG, Gülzow-Prüzen, Germany

**Keywords:** IGF-1, binding protein, bovine, pregnancy, gestation, embryonic mortality

## Abstract

Insulin-like growth factors (IGFs) play a critical role in fetal growth, and components of the IGF system have been associated with fetal growth restriction in women. In human pregnancy, the proteolytic cleavage of insulin-like growth factor binding proteins (IGFBPs), particularly IGFBP-4, releases free IGF for respective action at the tissue level. The aim of the present study was to determine IGFBP-2, IGFBP-3, and IGFBP-4 concentrations by Western ligand blotting during pregnancy until day 100 in cows and to compare these concentrations with those of non-pregnant cows and cows undergoing embryonic/fetal mortality. Therefore, two study trials (I and II) and an *in vitro* study were conducted. In study I, 43 cows were not pregnant, 34 cows were pregnant, and 4 cows were undergoing fm. In study II, 500 cows were examined, and 7 cases of pregnancy loss between days 24–27 and 34–37 after artificial insemination (AI, late embryonic mortality; em) and 8 cases of pregnancy loss between days 34–37 and 54–57 after AI (late embryonic mortality and early fetal mortality; em/fm) were defined from the analyses of 30 pregnant and 20 non-pregnant cows randomly selected for insulin-like growth factor 1 and IGFBP analyses. *In vitro* serum from pregnant (*n* = 3) and non-pregnant (*n* = 3) cows spiked after incubation with recombinant human (rh) IGFBP-4 for 24 h, and IGFBP-4 levels were analyzed before and after incubation to detect proteolytic degradation. The IGFBP-2, -3, and -4 concentrations did not decline during early pregnancy in cows, while IGFBP-4 concentrations were comparable between pregnant and non-pregnant cows, irrespective of low proteolytic activity, which was also demonstrated in cows. Interestingly, cows with em or fm showed distinct IGFBP patterns. The IGFBP-2 and -3 concentrations were higher (*P* < 0.05) in cows with fm compared to pregnant. The IGFBP-4 levels were significantly higher in cows developing fm. Thus, distinct differences in the circulating IGFBP concentrations could be associated with late embryonic and early fetal losses in cattle.

## Introduction

The somatotropic axis is a key regulatory pathway for dairy cows, and a growth hormone insensitivity plays a role in energy partitioning to facilitate milk production after calving (1). During lactation, a recoupling of the somatotropic axis occurs as milk yield decreases. The concentration of insulin-like growth factor-binding proteins (IGFBP)-2 and IGFBP-4 was shown to be elevated throughout the early lactation period (2). Dairy cows should achieve a new pregnancy within 2–3 months of calving in order to maintain high milk production. Therefore, the gestational period begins during lactation. Insulin-like growth factors (IGFs) play a crucial role not only for metabolic adaption but also in fetal growth. In this regard, the local IGF system, comprising insulin-like growth factors 1 and 2 (IGF-1 and IGF-2) and their respective IGFBPs are expressed in the endometrium prior to implantation and in the placenta (3–6). These are important as well as maternal IGF-1 and IGFBPs in the blood circulation. Here, we focus on maternal IGFBP concentrations because the bioavailability of IGF-1 in the blood is regulated by the binding of IGF-1 to six high-affinity binding proteins (IGFBP1-6) (7, 8). In the blood, the most abundant binding protein is IGFBP-3. This binding protein forms a complex with IGF-1 and the acid-labile subunit that restricts IGF-1 to the blood stream, representing an IGF-1 blood reservoir. The second most abundant binding protein in blood is IGFBP-2. The protein transports IGF-1 through the endothelium to cells expressing the respective IGF-receptors (IGFR) (8). Both IGFBP-3 and IGFBP-2 are mainly produced in the liver. IGFBP-4 is also detectable in serum and is described as an inhibitor of IGF activity (8, 9). In human pregnancy, the proteolytic cleavage of IGFBPs, particularly IGFBP-4, releases free IGF for respective action at the tissue level (10, 11). The responsible enzyme for IGFBP-4 proteolysis in women is pregnancy-associated plasma protein-A (PAPP-A), which is expressed in high amounts in the human placenta and released into the blood and, therefore, acts on maternal blood IGF-1 bioavailability (12). We previously showed that in heifers, the maternal IGFBP-4 concentration decreases towards day 18 of pregnancy and speculated that the proteolytic cleavage of binding proteins is the underlying reason for this decrease (13). However, whether proteolytic activity is present during pregnancy in cattle in maternal blood circulation, similar to humans, has not yet been investigated. Interestingly, high IGFBP-4 concentrations have previously been linked to fetal growth restriction in human gestation (14, 15), and less proteolytic cleavage might be due to higher IGFBP-4 and lower IGF-1 levels. In cows, also substantial differences in metabolic adaption throughout lactation may be responsible for differences in peripheral IGFBP-2 or IGFBP-4 concentrations and, therefore, play a role in pregnancy maintenance or serve as biomarker. Moreover, it was previously indicated in dairy cows that a higher peripheral IGFBP-3 concentration was associated with a lower fertility rate (16).

Therefore, the aim of the present study was to examine whether the maternal IGFBP concentration in blood decreases during early pregnancy in cows, to compare the IGFBP-4 concentration between pregnant and non-pregnant cows to examine cows undergoing embryonic/fetal mortality (fm) and to determine whether proteolytic activity for IGFBPs can be detected in pregnant cows.

## Materials and Methods

### Animals

To address the research questions, two subsequent experimental studies and *in vitro* testing were conducted. In the first experiment (Study trial I), pregnant and non-pregnant pluriparous cows from one farm were investigated until day 100 of pregnancy. In four animals, embryonic/fm occurred, and a specific IGFBP pattern was found in the blood of these animals. Therefore, a second sampling period (Study trial II) was utilized to compare the cattle suffering from em or em/fm among pregnant or non-pregnant cows. Further, we conducted *in vitro* analyses (*in vitro* trial) to determine whether the proteolytic degradation of IGFBP-4 is also detectable in cows.

### Study Trial I

Pluriparous Holstein Friesian cows from the Brunckhorst/Romundt GbR (Vahlde, Germany) were used. In total, 260 cows were maintained in a free-stall housing system with straw-bedded maternity pens. The study animals were selected out of 105 lactating and pluriparous animals (mean 305-milk yield of 9,000 kg). All the cows used for the present study had physiological calving without any dystocia or obstetrical assistance needed. The cows were fed a total mixed ration (consistent of corn and grass silage, grinded corn and wheat, beer marc, feed fat, minerals, lime, salt, and yeast) and provided free access to water and additional concentrate based on the daily milk yield *via* transponders. The study was performed in accordance with the German legislation on animal welfare (Lower Saxony Federal State Office for Consumer Protection 279 and Food Safety, AZ 33.9-42502-05-14A487).

#### Study design

Only pluriparous cows> 1 lactation were enrolled in the present study, and initially, the general health condition was examined. Subsequently, behavior, posture, body temperature, and body conditioning score (BCS) (17) were recorded. Additionally, the milk yield during the previous lactation was documented. A gynecological examination was performed to assess uterine health, and the cycle stage was determined *via* transrectal ultrasonography (HS 101V, HONDA Electronics, Tokyo, Japan). If the cows showed estrous symptoms (mounting, standing heat, mucus discharge), then day 0 was defined, and a blood sample was drawn from the coccygeal vessel directly into tubes with EDTA and in one tube without any anticoagulants to acquire serum (Sarstedt, Nümbrecht, Germany). The cows were subsequently artificial insemination (AI, day 0). On day 18 ± 1 after AI, the cows were gynecologically examined again, and a second blood sample was obtained. On day 42 ± 1 after AI, a pregnancy diagnosis was performed by transrectal ultrasonography and analysis of pregnancy-associated glycoproteins in blood samples. If the cows were diagnosed as pregnant, two subsequent blood samples were obtained, as described above (days 60 ± 3 and 100 ± 3). When pregnancy was clearly diagnosed on day 42 ± 1, but the cow was non-pregnant on either day 60 ± 3 or day 100 ± 3, then the animal was defined as undergoing fm. Therefore, three groups of animals were compared in this study trial (p, pregnant; np, non-pregnant; and fm, fetal mortality).

### Study Trial II

In total, 500 pluriparous Holstein Friesian cows from “Gut Schönerlinde/Wansdorf, Wandlitz” were examined four times after artificial insemination. The study was performed in accordance with the German legislation on animal welfare (state office of Brandenburg for work protection, consumer protection, and physical health; AZ 2347-29-2014). Estrous was detected by determination of estrous symptoms (mounting, standing heat, and mucus discharge) and additional rectal examination of the uterus and, if necessary, the ovaries following AI; this examination was performed by a herd insemination technician. After AI (days 10–14), the animals were clinically investigated, the BCS was documented, and blood samples were collected from the coccygeal vein. Subsequently, blood samples were collected on days 24–27 and 34–37 after AI, in all the animals and additionally on days 54–57 in pregnant cows. A pregnancy diagnosis was performed by using ultrasonography (My Lab^®^, Esoate, Italy) and analysis of PAG in the blood on days 24–27, 34–37, and 54–57. If on days 24–27, a pregnancy clearly was diagnosed but the cow was not pregnant on days 34–37, the cow was defined as late embryonic mortality (em). Blood samples were taken on day blood samples were collected on days 10–14, 24–27, and 34–37 but not on days 54–57. If on days 34–37, a pregnancy was clearly diagnosed but the cow was not pregnant on days 54–57, the animal was defined as late embryonic mortality or early fetal mortality (em/fm).

### Laboratory Analyses

#### Pregnancy-Associated Glycoproteins

Semi-quantitative PAG analysis was performed using a commercially available PAG-ELISA (IDEXX Bovine Pregnancy Test, IDEXX, Westbrook, ME, USA). The samples were analyzed according to the manufacturer’s instructions, and the optical density (OD) was interpreted with regard to Ref. (18). The PAG-ELISA is based on a system with an indirect sandwich ELISA, and the PAG OD was photometrically measured (SLT Spectra, SLT Lab Instruments GmbH, Salzburg, Austria).

#### Insulin-Like Growth Factor I

The total serum IGF-1 concentration was determined using a commercial RIA according to the manufacturer’s instructions (IGF-I IRMA A15729, Beckman Coulter, CA, USA), as previously described in cattle (19). This method is based on the “sandwich” principle, and the detection antibodies were mouse monoclonal antibodies against 2 epitopes of IGF-I. The intraassay CV was 5.1%, and the interassay CV was 9.3%.

#### Insulin-Like Growth Factor-Binding Proteins

**Figure 1:**
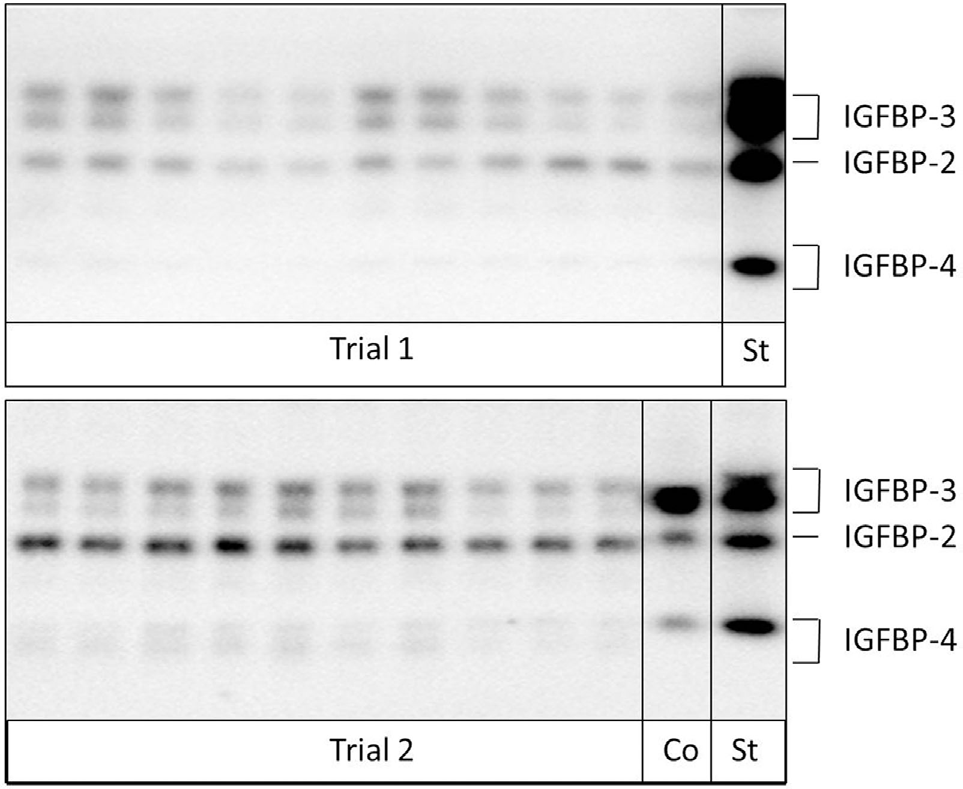
Analysis of insulin-like growth factor-binding proteins (IGFBPs) in samples from pregnant and non-pregnant cows by Western ligand blotting. Due to protein glycosylation, IGFBP-3 and -4 can be detected as band doublets (Co: internal control; St: human recombinant IGFBP-standard).

Quantitative Western ligand blotting of serum IGFBP-2, IGFBP-3, and IGFBP-4 levels was performed to detect the IGFBP concentration of functional binding proteins as described before (20). The lower and upper detection limits differed between the individual runs of BP analyses and were 195–12,500 ng/ml for IGFBP-2, 391–250,000 ng/ml for IGFBP-3, and 195–12,500 ng/ml for IGFBP-4 for Study trial I and 130–8,333 ng/ml for IGFBP-2, 260–16,000 ng/ml for IGFBP-3, and 260–16,666 ng/ml for IGFBP-4 for Study trial II. Figure 1 shows an example of a Western Ligand Blot of a pregnant and non-pregnant cow. All Western ligand blot results were normalized and presented as percent of the respective BP concentration of non-pregnant cows on day 0 (Study trial I) or days 10–14 (Study trial II).

#### *In Vitro* Protease Testing

Serum from pregnant (*n* = 3) and non-pregnant (*n* = 3) cows was diluted 1:10 with sterile phosphate-buffered saline, spiked with recombinant human (rh) IGFBP-4 (100 ng/µl) + rhIGF-1 (0.5 ng/µl) and incubated for 24 h at 39°C under soft agitation. Endogenous serum IGFBPs and rhIGFBP-4 were analyzed in duplicate as area under the curve using Western ligand blotting, as described in Section “Insulin-Like Growth Factor Binding Proteins”. Proteolytic degradation was detected before (A) and after incubation (B) using the value before incubation as 100%. As a positive control for the high proteolytic degradation of IGFBPs, human serum from a pregnant woman was available, which was treated similar to bovine serum. The human serum was available from one of the researchers, therefore, no ethical approval was necessary according to the ethics committee of the Hanover medical school.

### Statistical Analyses

Statistical analyses were performed using the statistic software R (21). Each variable was initially tested within the groups (p, np, em, and em/fm) for deviation from the normal distribution using the Shapiro–Wilk and Anderson–Darling tests. In some cases, the data differed from the normal distribution; however, due to the small sample sizes, standardized QQ-plots were additionally consulted to evaluate normality criteria. The QQ-plots displayed reasonable agreement of the sample quantiles with that from a standardized normal distribution; hence, parametrical tests were deemed appropriate. Additionally, the response variables were evaluated for the homogeneity of variances across these groups using the Brown–Forsythe test. Minor inhomogeneities of variances were tolerated due to the balanced study design and by applying a lower significance level. A two-factor analysis of variance (ANOVA) was conducted using the following model: *y* ~*x*1 + *x*2 + *x*1*x2, where *y* denotes the response variable of the measured traits and *x*1, *x*2 the explanatory variables group and time. Moreover, the interaction *x*1*x2 was considered in the model. If the ANOVA results for explanatory variables indicated a significant influence on the response variable, then further investigation by using a *post hoc* test (Tukey’s HSD) was conducted. A *P*-value of <0.05 was considered significant.

## Results

### Study Trial I

#### Animals

Out of 105 examined cows, 43 non-pregnant and 34 pregnant cows were enrolled in the blood sampling and analyses. In 3 cases, the cows were diagnosed as pregnant on day 60 and non-pregnant on day 100, and in another cow, pregnancy was lost between days 42 and 60 after AI. These four cases were defined as fm.

#### Laboratory Analyses

**Figure 2:**
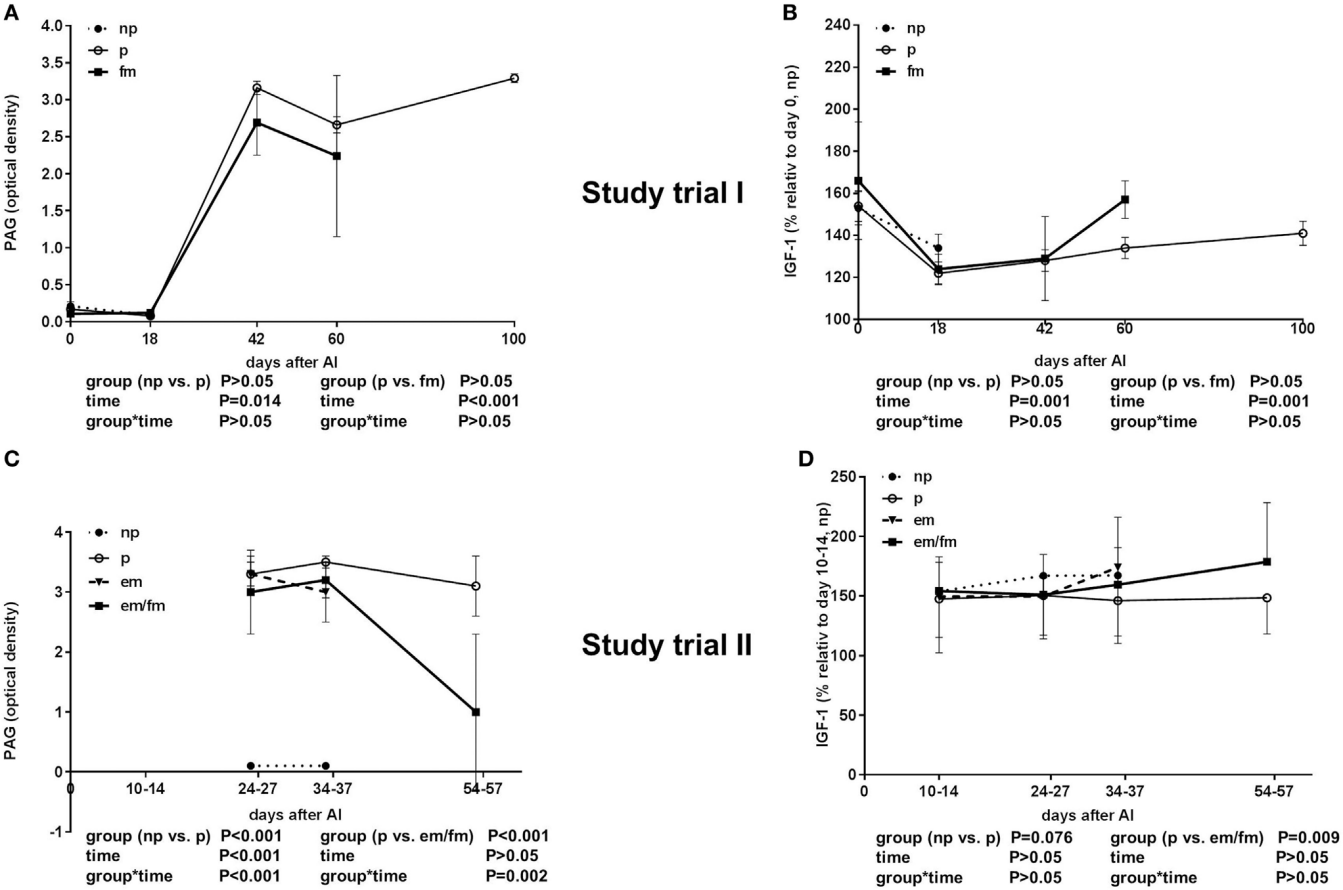
Semi-quantitative pregnancy-associated glycoproteins (PAG) values given in optical density (OD) units and insulin-like growth factor 1 (IGF-1) concentrations. **(A,B)** Study trial I in not pregnant [np, *n* = 43, day of AI (0) and 18 days after AI] and pregnant (p, *n* = 34, day of AI, and 18, 42, 60 and 100 days after AI) pluriparous Holstein–Friesian cows as well as cows with fetal mortality (fm, *n* = 4, day of AI and until day 60 after AI). **(C,D)** Study trial II in not pregnant (np, *n* = 20, 10–14 (IGF-1), 24–27, 34–37 days after AI) and pregnant (p, *n* = 30, 24–27, 34–37 and 54–57 days after AI) Holstein–Friesian cows as well as cows with embryo mortality (em, *n* = 7, 24–27, 34–37 days after AI) or late embryo/fetal mortality (em/fm, *n* = 8, 24–27, 34–37, and 54–57 days after AI). Values are given as mean ± SEM.

As expected, pregnant animals had high PAG levels until day 42 of pregnancy. The PAG values of pregnant and non-pregnant pluriparous cows were comparably low between days 0 and 18. There was no significant difference between pregnant cows and cows with fm; however, PAG levels were numerically lower in cows with fm than those in pregnant animals (Figure 2A).

The IGF-1 concentration was decreased in all the groups from days 0 to 18 and thereafter steadily increased from days 18 until 100 of gestation (*P* < 0.001). No differences between pregnant and non-pregnant cows, and cows with fm were detectable in the total IGF-1 concentrations; however, between days 42 and 60, an abrupt increase in the total IGF-1 in the cows suffering from fm was measured (Figure 2B).

**Figure 3:**
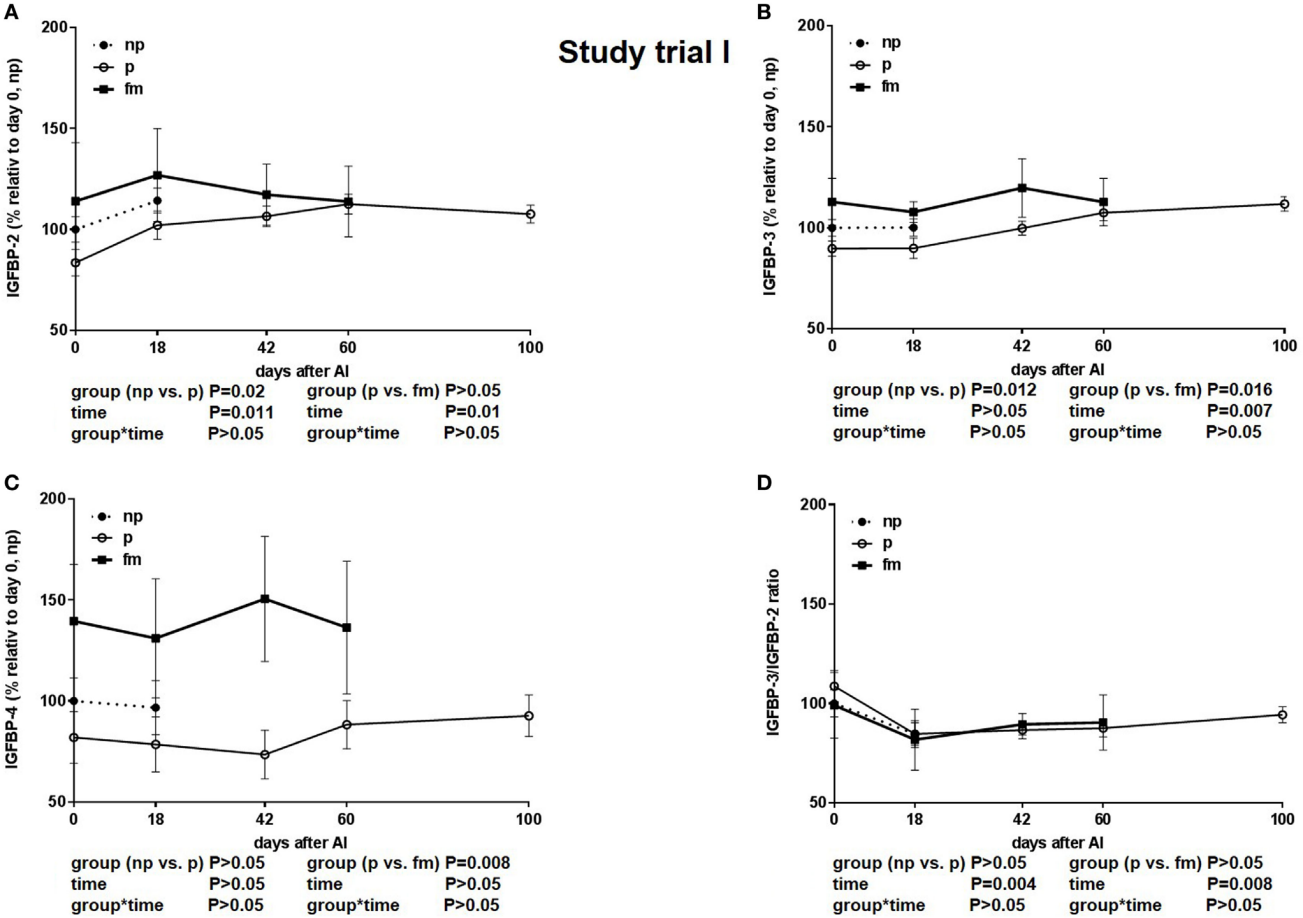
Study trial I: insulin-like growth factor-binding protein (IGFBP) **(A)**: -2, **(B)**: -3 and **(C)**: -4 concentration and **(D)**: IGFBP-3/IGFBP-2 ratio in not-pregnant [np, *n* = 43, day of AI (0) and 18 days after AI] and pregnant (p, *n* = 34, day of AI, and 18, 42, 60 and 100 days after AI) pluriparous Holstein–Friesian cows as well as cows with fetal mortality (fm, *n* = 4, day of AI and until day 60 after AI). Values are given in% relative to day 0 of non-pregnant cows. Values are given as mean ± SEM.

The IGFBP-2 concentration was lower in pregnant cows and highest in cows with fm. This distinct difference was visible on the day of insemination (Figure 3A). Additionally, the IGFBP-3 concentration was higher in cows with fm than that in pregnant cows (Figure 3B). IGFBP-4 was higher in cows with fm (*P* = 0.0076) if compared to pregnant and non-pregnant cows. Within the group of cows with fm, IGFBP-4 decreased from days 0 to 18 and thereafter increased until day 60 of pregnancy. In non-pregnant cows, IGFBP-4 decreased until day 42 after AI but increased later (Figure 3C). The IGFBP-3/IGFBP-2 ratio sharply decreased between days 0 and 18 and increased thereafter steadily until day 100 of pregnancy (Figure 3D).

### Study Trial II

#### Animals

Out of 500 examined cows, 203 were pregnant on days 24–27 after AI, and 284 were non-pregnant; in 15 cows with a positive pregnancy diagnosis on days 24–27 after AI, no pregnancy was detectable at either days 34–37 or 54–57 after AI. Therefore, two groups of pregnancy loss were defined: pregnancy loss between days 24–27 and 34–37 after AI = late embryonic mortality (em, *n* = 7) and pregnancy loss between days 34–37 and 54–57 after AI = late embryonic mortality and early fetal morality (em/fm, *n* = 8). A total of 13 animals were excluded from the study due to various illnesses. To analyze a balanced subset, *n* = 30 pregnant and *n* = 20 non-pregnant cows were chosen by random for IGF-1 and IGFBP analyses.

#### Laboratory Analyses

As expected, the PAG levels, displayed in OD, were high in pregnant animals and low in non-pregnant animals in Study trial II. The PAG OD decreased between days 34–37 and 54–57, clearly indicating pregnancy loss (Figure 2C).

**Figure 4:**
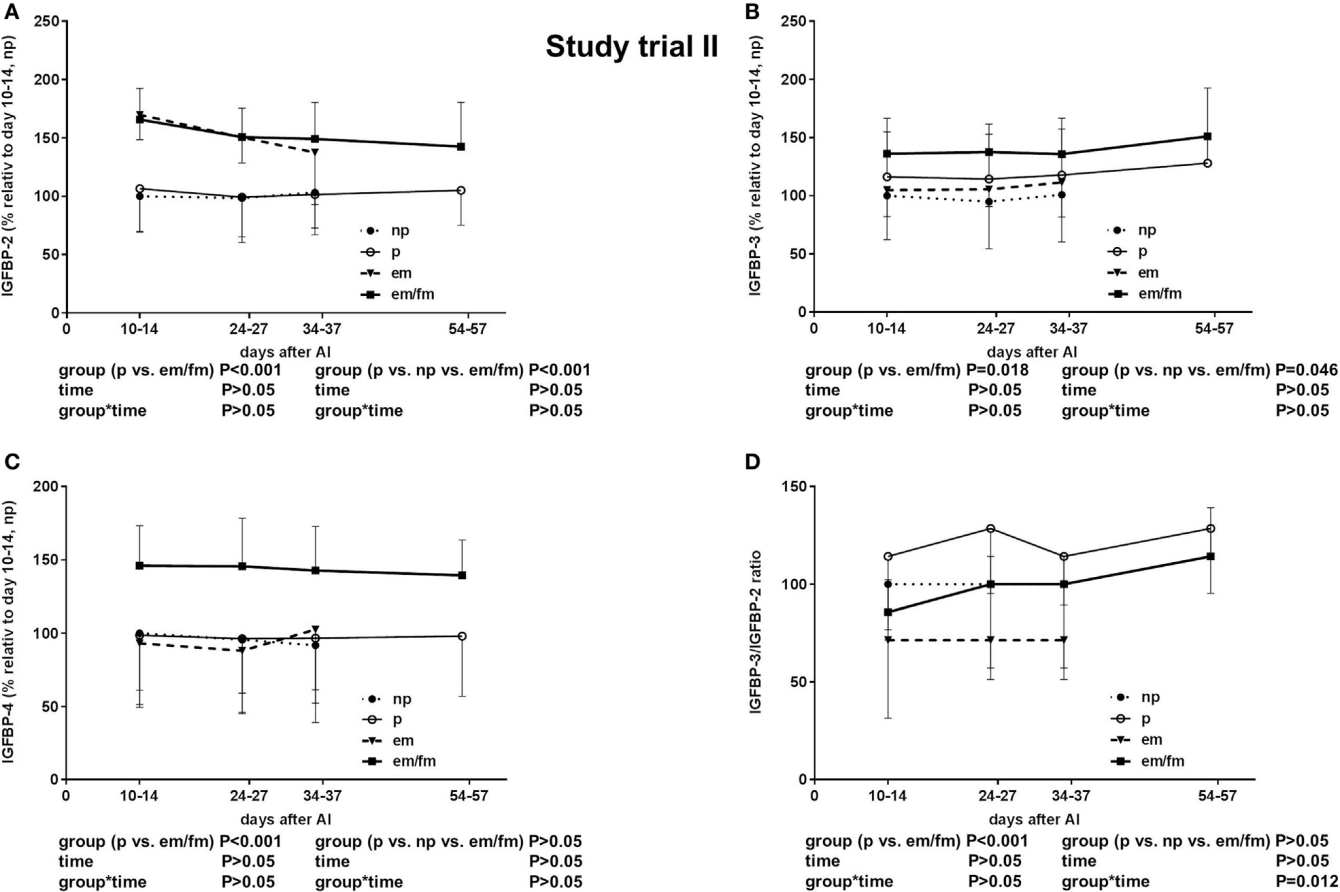
Study trial II: insulin-like growth factor-binding protein (IGFBP) **(A)**: -2. **(B)**: -3. **(C)**: -4 and **(D)**: IGFBP3/IGFBO2 ratio in non pregnant (np, *n* = 20) and pregnant (p, *n* = 30) Holstein Friesian cows and cows with embryo mortality (em, *n* = 7) between days 24-27 and 34-37 or late embryo mortality/early fetal mortality (em/fm, *n* = 8) between days 24-27 and 54-57. Values are given in percentege relative to days 10–14 of non-pregnant cows. Values are given as mean ± SEM.

The total IGF-I concentrations were tended to be lower in pregnant cows if compared to non-pregnant cows or cows with em (*P* = 0.0076; Figure 2D). The IGFBP-2 concentration was significantly higher in cows with em and em/fm compared to pregnant and not pregnant cows (Figure 4A). IGFBP-3 differed between pregnant and cows with em/fm. The latter had higher IGFBP-3 concentrations than did pregnant and not pregnant cows (Figure 4B). IGFBP-4 was clearly higher in cows with em/fm compared to the other groups (Figure 4C). The IGFBP-3/IGFBP-2 ratio was clearly lower in cows with em/fm than in pregnant cows (Figure 4D).

#### *In Vitro* Trial

**Figure 5:**
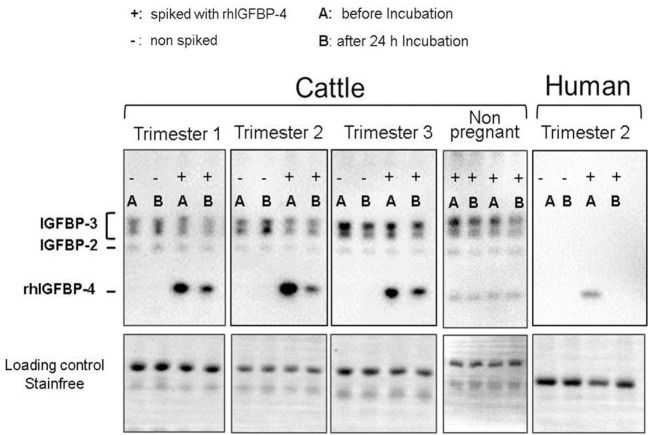
Western ligand blots with serum samples of not-pregnant and pregnant cows before (−I) and after (+I) incubation (24 h, 39°C) with Insulin-like Growth factor 4 (IGFBP-4) and insulin-like growth factor 1 (IGF-1) spiked to the serum. As positive control serum of a pregnant woman was available for which proteolytic activity IGFBP-4 is clearly proven.

The proteolytic degradation of rhIGFBP-4 was detected in pregnant cows (25–75% reduction of IGFBP-4) in all trimesters. The rhIGFBP-4 was 100% degraded in human serum. No proteolysis of rhIGFBP-4 was detected in non-pregnant cows. Moreover, no proteolysis of endogenous serum IGFBP-2 and IGFBP-3 in pregnant cows was obvious compared to that in the human control, where nearly all binding proteins were absent during pregnancy (Figure 5).

## Discussion

Both study trials were independently conducted in 2015 and 2016 on two different farms with different animals. Therefore, a farm-specific influence on the results comparably obtained in the two study trials can be excluded, thereby strengthening the results. In Study trial I, 4 pluriparous cows with fm were defined (3 between days 60 and 100 and 1 between days 42 and 60). In Study trial II, a higher number of animals (*n* = 500) was examined; therefore, 8 cows were found with late embryonic mortality (between 24–27 and 34–37), and 7 cows were found with late embryonic/early fm (between 34–37 and 54–57). The early fetal losses (between 42 and 60, *n* = 1) of Study trial I were most comparable to those of late embryonic/early fm (between 34–37 and 54–57) in Study trial II. The late losses in study I (>day 60) were not comparable to those in Study trial II, but the later fetal losses >day 42, which were not infectious abortions, might be due to placental dysfunction, fetal undernourishment or insufficient growth and, therefore, may be associated with IGF systems. However, no standardized microbiological or viral examination was done. Therefore, this assumption remains speculative.

In both studies, fetal loss was indicated not only by the empty uterus during ultrasound examination but also by declining PAG concentrations in the blood. PAGs are produced by binucleate cells of the placenta, indicating functional placentation (22, 23). Decreasing concentrations are associated with declining placenta function and pregnancy loss.

The local IGF system in the endometrium is crucial for embryonic development and is influenced by the cycle stage (24) but little is known about the role of the IGF-1 reservoir in blood in embryonic and fetal development or placental growth in cattle. IGF-1 is bound to specific binding proteins that indeed regulate either the transport of IGFs through the endothelium (IGFBP-2) or the retention in the blood stream (25). Specific proteolysis of the binding proteins in maternal blood can release IGF and may also enhance the free concentration at the tissue layer. Therefore, the aim of the present study was to characterize IGFBPs throughout early pregnancy in maternal blood circulation and compare those to either non-pregnant cows at the same cycle stage or to those suffering from embryonic or fm. Thus, embryonic mortality is defined as a pregnancy loss before day 42 of pregnancy in cows, and thereafter, the fetal period starts (26).

The total IGF-1 concentration in pregnant cows decreased from days 0 to 18 in the present study, typically due to sexual steroid hormone patterns during the cycle (19). This effect was previously observed by the application of estradiol and progesterone to ovariectomized heifers (27). In the first trimester of pregnancy (days 42–100), in Study trial I, a slight but steady increase in IGF-1 was measurable, whereas the concentrations remained relatively constant until day 60. However, the IGF-1 concentration was comparable between pregnant and non-pregnant cows and cows with embryonic/fm in both study trials. This finding is consistent with those of previous studies in Angus cattle, where no differences in pregnancy rates were found among animals selected for different IGF-1 concentrations (28). Consistently, in a recent study, the basal GH concentration was associated with the ability of cows to achieve pregnancy (29), but not total IGF-1 concentrations. In contrast to *in vivo* results, *in vitro* studies clearly demonstrated that IGF-I has a positive effect on embryo development (30–32). Indeed, *in vitro* embryos treated with IGF-1 during *in vitro* culture achieved a higher pregnancy rate after embryo transfer (28, 29). The conflicting *in vitro* and *in vivo* results might be due to IGFBPs, which on the one hand can be produced locally by the placenta but also the maternal IGFBPs might have an influence on the free IGF-1 concentration, which may have an influence on placental or fetal growth. The total IGF-1 concentration, also measured in the present study, does not reflect the bioavailability of IGF-1 on the local tissue level. The bioavailability differs with respect to which binding protein IGF is bound and the concentration of these single binding proteins. As in other species, distinct changes in IGFBP concentrations were previously measured throughout pregnancy, and their vital roles have been suggested (33). Interestingly, in women, there is a strong proteolysis of IGFBPs (IGFBP-2 and IGFBP5) modulated by the expression of PAPP-A in the placenta, resulting in high levels of circulating PAPP-A in pregnant women during the first trimester (10, 34). Moreover, a high level of PAPP-A could be detected in the human trophoblast. IGFBP-4 is described to be proteolysed as well by PAPP-A (35).

The present study examined the peripheral IGFBP-2, IGFBP-3, and IGFBP-4 concentrations by using a Western ligand blot technique, which enables the detection of functional binding proteins in serum (20). In contrast, antibody based methods might also detect binding protein fragments and, therefore, might not necessarily correlate with the biological binding capacity of IGFBPs.

In both study trials, pregnancy in cows did not distinctly influence the IGFBP-2 concentration over time. Although, PAPP-A is described in bovine preovulatory follicles and there able to degrade IGFBP-2 (36). However, to the best of the authors’ knowledge, there is no focused study on placental PAPP-A in bovine species. Interestingly, IGFBP-2 levels were higher in cows developing fm during the first 60 days of pregnancy than those in cows with physiological pregnancies. This fact was observed in both study trials consistently, specifically at days 0 and 18 in Study trial I and days 10–14 in Study trial II. IGFBP-2 is considered an activator and inhibitor of IGF action and is known as a main serum carrier of IGF-1 despite of IGFBP-3 (37). Therefore, increased concentrations may also inhibit IGF-1 action, and thus, influence fetal or placental growth. The association between fertility and this particular binding protein has previously been demonstrated by Wathes (38), who examined SNPs in the IGFBP-2 gene associated with a lower rate of pregnancy in cows with a specific SNP in this gene. Moreover, hepatic IGFBP-2 production was also directly controlled by growth hormone (37), which might explain the results of the above-mentioned study, where GH, but not IGF-1, is directly linked to pregnancy success. Under conditions of elevated, IGFBP-2 expression inhibitory effects have been provided both on growth and reproductive development (39). In women, the IGFBP-2 concentration declined after conception and began to steadily increase thereafter (33). Interestingly, elevated IGFBP-2 in the maternal blood circulation has been associated with growth restriction due to placental dysfunction in human medicine (38). In the present study, a comparable pathomechanism might be involved, as the pregnancies were lost after conception and placentation, suggesting that placental dysfunction and fetal growth retardation might be involved in fm. In the present study, ultrasound was conducted to detect pregnancy, but in a routine manner. Thus, there is no information on fetal length available, but this measurement should be considered in further studies. On the other hand, also the metabolic adaption throughout early lactation may lead to the peripheral IGFBP-2 concentrations as it was indicated that during early lactation IGFBP-2 was higher and decreases over the lactation period (2).

In human pregnancies, the complete proteolysis of IGFBP-3 was previously observed (40), and it was also observed in the human control serum in the present *in vitro* trial. It has to be mentioned that the human control was from a twin pregnancy and, therefore, likely have a higher proteolytic potential than in singleton human pregnancies (41). However, in cows, there was no drastic decline in IGFBP-3 throughout pregnancy until day 100, but rather an increase was measured until day 100 of pregnancy. Also *in vitro* there is still the IGFBP-3 band detectable.

In addition to the elevated IGFBP-2, IGFBP-3 was also higher in cows developing fm; this finding was also observed already during early embryonic development around days 10–18 after insemination. IGFBP-3 is the most abundant binding protein, and 80–90% of IGFs are bound to IGFBP-3 (38); in human pregnancy, the proteolysis of binding proteins, particularly IGFBP-3 (42), shifts the control of IGF action to IGFBP1, which offers the highest affinity to IGF-1 (43). The present study (Trial I) indicated that in cows, IGFBP-3 concentrations increased toward day 100 of pregnancy. Western ligand blotting only detects functional binding proteins; therefore, it is unlikely that single proteolytic fragments individually bind IGF-1, resulting in increased concentrations. Therefore, the mechanism controlling fetal and placental growth by the maternal somatotropic axis appears to be different between bovine and human. Increased concentrations of IGFBP-3 may indicate that less IGF-1 is free for local action. In future studies, the additional measurement of free IGF-1 (in blood and local) should be contemplated in order to answer this question.

One of the main goals of the present study was to measure the concentration particularly of IGFBP-4, as inhibitory binding proteins previously associated with fetal growth restriction. In early physiological pregnancies in women, an increase in IGFBP-4 was measured, followed by lower concentrations (15). We previously indicated a decrease in IGFBP-4, also measured by Western Ligand blot (13). In contrast to these results, no specific decrease in IGFBP-4 was found in cows in the present study in pluriparous cows. One difference between these both studies was that Meyerholz et al. (13) used heifers exclusively whereas in the present study mainly pluriparous, meaning lactating animals, are examined. This could be the underlying reason for differences in peripheral IGFBP pattern.

Oxvig (12) summarizes the importance of PAPP-A within the IGF-1 system and concluded that PAPP-A is an important component of the IGF system and describes this part of the IGF system as the “PAPP-A→IGFBP-4→IGF axis.” In cattle, PAPP-A has also been described in the context of follicular growth as already mentioned above, where preovulatory ovarian follicles are characterized by a high proteolytic cleavage of IGFBP-4 through PAPP-A (44). Moreover, variants in the PAPP-A gene were associated with “daughter pregnancy rate” but no detection during gestation in cattle was reported so far. In the present study, in pregnant cattle, *in vivo* data suggest that no distinct cleavage of IGFBP-4 occurs in bovine pregnancies as the concentrations stay comparable. But, *in vitro* a degradation of IGFBP-4 could be verified, however, no proof of the responsible enzyme e.g., PAPP-A was intended and should be the focus of further studies. Interestingly, in both study trials, IGFBP-4 was clearly lower in physiological pregnancies than in those with fm. Thus, we speculate that a high concentration of an inhibitory binding protein in maternal blood may be associated also with lower free IGF levels, which might have an important influence on fetal growth or placentation or that this high concentrations just mirrors a sub function of local proteases also released in the maternal blood circulation. Moreover, also the metabolic adaption of the dairy cow with regard to parallel lactation maybe lead to higher IGFBP-4 concentrations (2). Therefore, further studies are warranted to elucidate the underling pathomechanism for higher IGFBP-2- and IGFBP-4 concentrations in cows with pregnancy loss.

In conclusion, distinct differences in the peripheral IGFBP pattern could be associated with late embryonic and early fetal losses in cattle. Specific IGFBP-4 proteolysis could be identified in cattle but less intense than in human as peripheral concentrations were comparable between pregnant and not pregnant animals. Therefore, circulating concentrations of IGFBP-2, IGFBP-3, and IGFBP-4 in the mother may have a biomarker potential for maintenance of pregnancy in dairy cows.

## Ethics Statement

The study (Trial I) was performed in accordance with the German legislation on animal welfare (Lower Saxony Federal State Office for Consumer Protection 279 and Food Safety, AZ 33.9-42502-05-14A487). The study (Trial II) was performed in accordance with the German legislation on animal welfare (state office of Brandenburg for work protection, consumer protection, and physical health; AZ 2347-29-2014). The human serum was available from one of the researchers, therefore no ethical approval was necessary according to the ethics committee of the Hanover medical school.

## Author Contributions

MK: organization Study trial II, sampling and examinations for Study trial II and manuscript proof reading. H-DJ: organization and sampling for Study trial I. WE and BY: *in vitro* tests and writing of material and methods as well as results for the *in vitro* test. BR: statistical analyses. PS: sampling and examination for Study trial II and manuscript proof reading. JM: organization of Study trial II and manuscript proof reading. HC: western ligand blotting. HA: interpretation of data and manuscript proof reading. SM: coordination of the study, writing the manuscript, and interpretation of data.

## Conflict of Interest Statement

HC and HA are related to Ligandis. All other authors state that there are no conflicts of interest to declare.
